# Removal of a Large Scirrhous Testicle from a Man While under the Influence of Nitrous Oxide Gas

**Published:** 1847-10

**Authors:** E. E. Marcy

**Affiliations:** Hartford, Conn.


### SELECTIONS

FROM

### AMERICAN AND FOREIGN JOURNALS.

---

*Removal of a large Scirrhous Testicle from a Man while under the Influence of Nitrous Oxide Gas.* By E. E. Marcy, M.D., of Hartford, Conn.—The subject of the operation was a young man, 24 years of age. He had been afflicted with an enlargement of the testicle for about a year past. Within the last few weeks the disease progressed so rapidly that the lower portion of the gland and scrotum became gangrenous and sloughed. The case was highly unfavorable in every respect, yet believing extirpation to be the only means which could save the man's life, the operation was performed on the 17th of August, the protoxide of nitrogen having been previously administered by Dr. Wells, the discoverer. The patient commenced inhaling the gas at half past 1 o'clock, P.M., and after about one minute from this time the operation was commenced. At the first incision there was a slight manifestation of pain (the full effect of the gas not having yet been received), but from this instant until the diseased mass was removed, and all the bloodvessels secured (there being quite a number which required ligatures), there was not the slightest consciousness of pain on the part of the patient. We were satisfied that this was the fact during the operation, from the placid and happy expression of his countenance, from the entire absence of all muscular efforts, and from the natural and unexcited state of the pulse (this having remained without any apparent variation during the whole period). The operation was necessarily tedious and protracted on account of the great size of the gland, the extensive and firm adhesions of the integuments to the diseased structure, and the unnatural enlargement of several arteries which required ligature. The whole period consumed, from the commencement of the operation until the vessels were secured, was not far from fifteen minutes. On questioning the patient afterwards, he asserted that he experienced a *slight* painful sensation at the commencement of the first incision, but from that time until the dressings were applied he was entirely unconscious of any pain!

After the operation, he expressed himself as feeling perfectly well, except some smarting in the wound; no pain or other unpleasant feeling in the head or any other part of the body; pulse regular and natural, as before the operation.

August 18th. Since the operation, the patient has suffered no pain or other unpleasant symptoms. Pulse 82, and moderately firm. Expresses a strong affection for the gas-bag, and an earnest desire to retain it in his possession as the grand balm for the pains and troubles of this life.

The above case affords additional testimony (if this was required) that the nitrous oxide is capable of banishing sensibility in the most severe operations, and that, too, without exposing the patient to any of the untoward effects which result from the use of ether. The latter article exerts a more deleterious effect upon the nervous system than the former, as is indicated by the pain in the head, lassitude, &c., which follow its use. Another still more important objection to the use of ether, arises from its injurious effect upon the blood. It has been found by experiment that the arterial blood becomes highly charged with carbon after the inhalation. The effect of this upon the system must be very injurious; for unless the due proportion of oxygen be retained in the arterial blood, diminished nervous force and vital energy, with other states which *at least* predispose to disease, must be induced.

The above objection will not hold good in relation to the nitrous oxide, as its constituents are the same as common air with an increased proportion of oxygen; while the ether bears no analogy to the air, and will therefore be more prone to give rise to injurious consequences. The effect of ether upon the circulatory vessels is in the first instance extremely violent, succeeded by an alarming state of depression in their action. The effect of the gas is much milder upon these vessels, and never need be carried to such an extent as to be followed by any depression.

When Dr. Wells made the great discovery, in 1844, that the inhalation of nitrous oxide gas would render the body entirely insensible to the pain of surgical operations, the question suggested itself to me, as well as some others, of this city, whether sulphuric ether might not answer as good a purpose as the gas. This subject was fully discussed at the time by a number of professional men here, and a trial made with the ether; but the general opinion was then formed, that the nitrous oxide was on many accounts preferable. Numerous trials with both these substances, from that period to the present time, have demonstrated conclusively that this opinion was correct.

I am informed by Dr. J. M. Riggs, of this city, that he has used the gas constantly since November, 1844, and with uniform success. He has performed more than one hundred dental operations on patients while under its influence, and with more uniform success than has resulted from the use of the ether.

Dr. Wells has used the gas in only about fifty instances, on account of his relinquishing his professional business for a time. We are assured by both these gentlemen, that in no instance have they been troubled by muscular efforts on the part of their patients. Indeed, it may be asserted with safety, that so far as muscular action is concerned, it possesses a decided advantage over the ether. We are aware that it has been impudently asserted by certain interested persons, who have never given the protoxide a trial in an operation, that the patient will become "dancing mad," &c., &c. But facts prove this to be far from the truth. So far, then, the gas is preferable to the ether.

Another superiority which it possesses over the ether, is that its after-effects are far less unpleasant—less headache, less lassitude, and less depression of the nervous system, always resulting from its use. Ether generally causes troublesome choking and cough; the gas scarcely ever. Ether is objectionable on account of the unpleasant smell which it communicates to the room; the gas possesses no disagreeable odor. Ether abstracts largely from the oxygen of the arterial blood, thus becoming a direct source of disease; the gas has no such effect. Ether gives rise to pains in the head, lassitude, impaired vital energy, and other symptoms indicating serious depression of the nervous system; the gas rarely produces any of these effects, and if *ever*, only in a slight degree. In order to produce the full effect of the ether, it is customary to reduce the patient to a state of stupor; the gas is capable of rendering the body entirely insensible to the pain of the most severe surgical operation, without putting the patient to sleep, or causing any stupor! We have often observed patients watch the progress of severe operations upon their own persons, with countenances as smiling and happy as if they were enjoying a delightful treat.

We firmly believe that the gas would have long since entirely superseded the use of the ether, had it not been for the trouble attending its preparation. We trust, however, that in future this slight inconvenience will not prevent the surgeon, who has the welfare of his patient at heart, from making use of the agent so manifestly superior in its effects.

The State Legislature of Connecticut, which has just closed its session, has, after a due consideration of the evidences, fully recognized Dr. Horace Wells, of Hartford, as the sole discoverer, and have passed him a vote of thanks for this great discovery, which consists, as the vote expresses it, in the use of "nitrous oxide gas or ether in surgical operations." Thus the question of priority is finally settled by legislative enactment.—*Boston Med. and Surg. Jour.*

